# Efficient computation of spaced seeds

**DOI:** 10.1186/1756-0500-5-123

**Published:** 2012-02-28

**Authors:** Silvana Ilie

**Affiliations:** 1 Department of Mathematics, Ryerson University, Toronto, ON M5B 2K3, Canada

**Keywords:** Similarity search, Local alignment, Spaced seed, Heuristic algorithm, Sensitivity

## Abstract

**Background:**

The most frequently used tools in bioinformatics are those searching for similarities, or local alignments, between biological sequences. Since the exact dynamic programming algorithm is quadratic, linear-time heuristics such as BLAST are used. Spaced seeds are much more sensitive than the consecutive seed of BLAST and using several seeds represents the current state of the art in approximate search for biological sequences. The most important aspect is computing highly sensitive seeds. Since the problem seems hard, heuristic algorithms are used. The leading software in the common Bernoulli model is the SpEED program.

**Findings:**

SpEED uses a hill climbing method based on the overlap complexity heuristic. We propose a new algorithm for this heuristic that improves its speed by over one order of magnitude. We use the new implementation to compute improved seeds for several software programs. We compute as well multiple seeds of the same weight as MegaBLAST, that greatly improve its sensitivity.

**Conclusion:**

Multiple spaced seeds are being successfully used in bioinformatics software programs. Enabling researchers to compute very fast high quality seeds will help expanding the range of their applications.

## Background

The most frequently used tools in bioinformatics are those searching for similarities, or local alignments, between biological sequences. This problem can be solved exactly using the dynamic programming algorithm of Smith-Waterman in quadratic time. Many instances, including all database searches, are too large for this approach to be feasible and heuristic algorithms are used instead [1,2]. The most widely used program in bioinformatics, BLAST [2,3], is one such tool. It uses the so-called "hit and extend" approach: a hit consists of 11 consecutive matches between two sequences and represents a potential local alignment. The hit is then extended both ways in search for similarity.

It is clear that not all local alignments have to include an identical stretch of length 11. It has been already noticed in [4] and then again in [5] that requiring that the matches are not consecutive increases the chances of finding alignments. The idea of optimizing the way the required matches are placed has been investigated in [6,7], the latter having used it in a similarity search software, PatternHunter. Much work has been dedicated to spaced seeds. For a survey of earlier work, we refer the reader to [8].

The 11 consecutive matches of BLAST are called a *contiguous seed*, denoted 11111111111 (for 11 consecutive matches), whereas the one of PatternHunter is a *spaced seed*, 111*1**1*1**11*111; a 1 represents a match and * a don't care position. The number of 1's represents the *weight *of the seed. The probability of finding local alignments, under specific conditions, to be made precise later, is called *sensitivity*.

We notice an essential trade off. Decreasing the number of matches, that is, the weight of the seed, increases the sensitivity but also the number of random hits, decreasing specificity. On the other hand, it is intuitively clear that several different seeds will hit different alignments, thus having increased sensitivity. It has been noticed by [9] that doubling the number of seeds can account for the decrease in weight, thus simultaneously increasing sensitivity without reducing specificity. PatternHunterII [9] uses 16 different seeds of weight 11. For comparison, under similar conditions, the sensitivity of the BLAST, PatterHunter, and PatternHunterII seeds, all of weight 11, are 0.30, 0.47, and 0.92, respectively, a very large difference.

Multiple spaced seeds represent the current state of the art in similarity search and are used by many software programs, in a variety of applications, such as sequence alignment [6,9,10], read mapping [11,12], or oligonucleotide design [13]. It is therefore of great importance to be able to compute seeds with high sensitivity. The only way to find optimal seeds seems to be by trying all possible ones. This brute force approach includes two exponential steps. First, there are exponentially many candidates. Second, computing sensitivity is exponential as well. Therefore, only single seeds can be computed this way. For multiple seeds, since the relevant problems are hard [14,15], heuristic algorithms must be used. Among many such algorithms, such as Mandala [16] and Iedera [17], only one works in polynomial time: SpEED [18]. SpEED is based on the notion of *overlap complexity *[19], that is very well correlated with sensitivity but polynomial-time computable. A hill climbing algorithm is used that iteratively swaps a 1 with a * in a random seed in order to improve the overlap complexity.

Our contribution in this paper is to improve the best existing software, SpEED, by increasing its speed and, consequently, the sensitivity of the computed seeds. The first algorithm we give is a bit-parallel algorithm for computing overlap complexity. This is of independent interest and alone can speed up the hill climbing of SpEED significantly. However, we give a better algorithm for this heuristic that improves its speed by one order of magnitude. Several tests are provided to prove these claims. Then, the new implementation is employed to compute improved seeds for PatternHunterII as well as BFAST, as they use some of the most demanding seeds. Finally, we show a very significant improvement of the MegaBLAST seeds. At weight 28 they are significantly larger than everything else, yet we manage to compute up to 16 seeds of this weight, with very large improvement in sensitivity over MegaBLAST.

### Spaced seeds

A *spaced seed *is any string containing 1's and *'s. Since having a * at one end of a seed is not useful, we assume that all seed start and end with a 1. For a seed *s*, the weight *w *of *s *is the number of 1's and the *length *ℓ is the number of all letters. The *i*th letter of *s *is denoted *s*[*i*]. A multiple spaced seed is a set of seeds *S *= {*s*_1_, *s*_2_, ..., *s*_*k*_}. In the Bernoulli model [9] an alignment is represented as a (random) sequence *R *of 1's and 0's (matches and mismatches) where the probability *p *of a match is called *similarity*. The length *N *of this region *R *plays an essential role in the sensitivity. We say that a seed *s hits R *if there is a position *i *in *R *such that, for any *j*, 0 ≤ *j *≤ ℓ-1, if *s*[*j*] = 1, then *R*[*i *+ *j*] = 1. That means, aligning *s *with *R *starting at position *j *causes all 1's in *s *to correspond to 1's in *R*. This definition extends naturally to multiple seeds: *S *hits if one of its seeds does so.

The *sensitivity *of *s *(or *S*) is then defined as the probability that *s *(or *S*, respectively) hits *R*. It depends on the distribution of matches in the seed as well as on the length *N *of the region to be hit and the similarity level *p*. The sensitivity can be computed by the dynamic programming (exponential) algorithm given in [9].

### Overlap complexity

Since the number of expected seeds is proportional to the weight of a seed, it is fair to compare seeds of the same weight. Thus, given two seeds of weight *w*, one contiguous and one spaced, the spaced one may have higher sensitivity because its hits do not overlap as much and are therefore better distributed, covering more similarities. The distribution of the 1's is nevertheless crucial for the quality of the seed. For instance, periodically spaced seeds are worse than contiguous. The problem of finding optimal seeds seems difficult and heuristic algorithms are employed. The only polynomial-time algorithm, implemented in SpEED [18], is based on the notion of *overlap complexity *[19], that captures the amount of overlaps between hits. Given two seeds *s*_1 _and *s*_2_, for each of the possible |*s*_1_| + |*s*_2_|-1 overlaps between them, denote by σ_*i *_the number of overlapping positions where both seeds have a 1.

**Figure 1:**
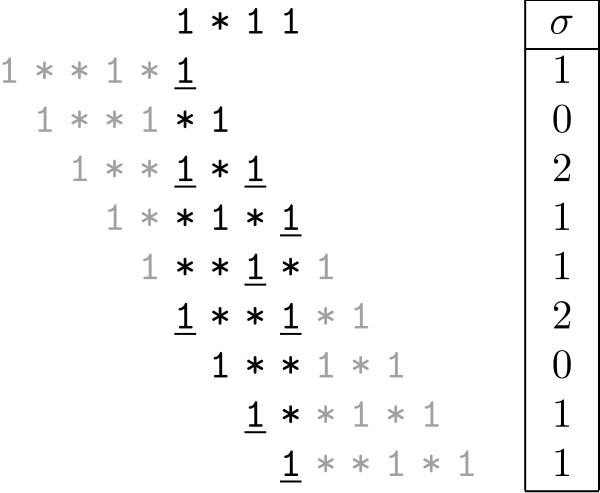
**Overlap complexity example**. An example of the overlap complexity between two spaced seeds. Letters not taking part in the overlaps are grey and the overlapping pairs of 1's are underlined; the values of σ for each overlap are given in the last column

The overlap complexity is defined as ${\text{OC}}_{\left(s_{1},s_{2}\right)}=\sum_{i}2^{\sigma_{i}}$. The overlap complexity of any seed *S *= {*s*_1_,*s*_2_, ...,*s*_*k*_} *is *defined by: $\text{O}{\text{C}}_{\left(S\right)}=\sum_{i\leq j}\text{O}{\text{C}}_{\left(s_{i},s_{j}\right)}$ As an example, $\text{O}{\text{C}}_{\left(1*11,1**1*1\right)=2^{1}+2^{0}+2^{2}+2^{1}+2^{1}+2^{2}+2^{0}+2^{1}+2^{1}=20}$. The overlaps are shown in Figure 1.

A very strong experimental correlation between overlap complexity and sensitivity has been observed in [19]. Seeds with low overlap complexity have high sensitivity. The algorithm of [18] employs a hill climbing algorithm to constructs highly sensitive seeds. Iteratively, (1, *) pairs are swapped to reduce the overlap complexity of a random seed (see [18] for details). The SpEED software runs much faster and produces better seeds than all the other programs.

### Faster overlap complexity

**Figure 2:**
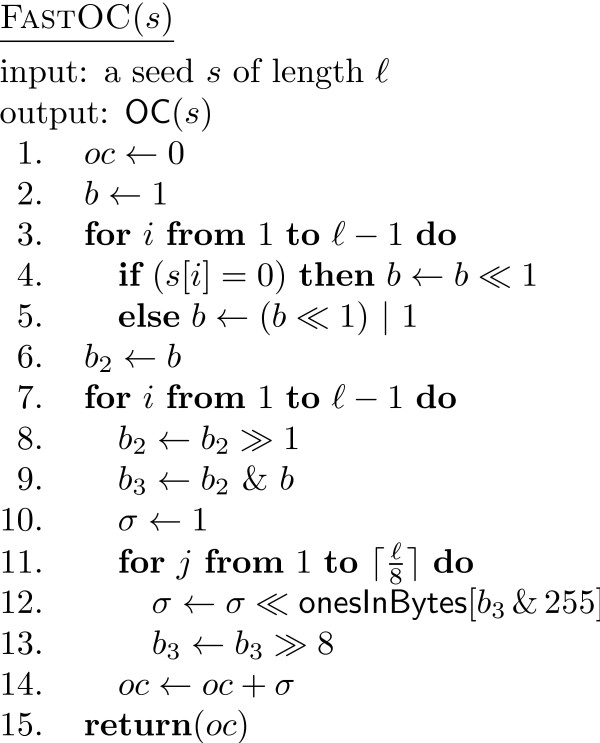
**The pseudocode of FastOC**. The pseudocode of the new faster implementation for overlap complexity computation, FastOC

Our first algorithm computes the overlap complexity faster than the one in [18,19]. It first converts each seed into 64-bit integers by interpreting 1 and * as bits 1 and 0, respectively. For instance, 1**11 is converted into the integer 10011 = 19. The overlap between two seeds is then computed by shifting the bits and AND-ing the integer representation of the seeds. In order to compute the number of 1's in an integer, we assume a precomputed array onesInBytes with 256 components such that onesInBytes[*i*] given the number of 1's in the binary representation of *i*. Then, the number of 1's in a 64-bit integer is computed by iteratively shifting right 8 positions, AND-ing with 255 and then using the onesInBytes array. The pseudocode (procedure FastOC) is given in Figure 2. For simplicity, the computation of the overlap complexity of a single seed is shown. It extends immediately to any multiple seed.

A slightly faster algorithm is obtained by using a precomputed onesInTwoBytes array that stores the number of 1's in the binary representation of 16-bit integers, that is, between 0 and 65536. We shall call this algorithm VFastOC. We have compared the FAstOC and VFastOC algorithms with the original one implemented in SpEED in Table 1. The VFastOC algorithm is the fastest, its running time being up to four times lower than the original. The algorithm VFastOC is of interest in itself and also replacing the original one with VFastOC would improve the speed of the heuristic correspondingly. However, we give a better solution in the next subsection.

**Table 1:** Comparison of overlap complexity computation algorithms

*w*	ℓ	OC	FastOC	VFastOC
9	15	0.012	0.004	**0.004**

10	17	0.064	0.028	**0.024**

11	18	0.116	0.048	**0.044**

12	19	0.204	0.084	**0.072**

13	20	0.340	0.136	**0.116**

14	21	0.564	0.208	**0.184**

15	23	2.792	0.948	**0.828**

16	24	4.564	1.484	**1.300**

17	25	7.276	2.648	**1.992**

18	26	11.368	3.968	**2.984**

Speed comparison between the existing and the new implementations of the overlap complexity function. Seeds of optimal length for weights between 9 and 18 are considered. In each case, the time (in seconds) is given for the computation of overlap complexity for all $\left(\begin{array}{c} \ell \\ w \end{array}\right)$ seeds with the given parameters. The VFastOC algorithm is the fastest (times in bold), up to 3.8 times faster than the original OC algorithm.

### Faster hill climbing

The heuristic of SpEED uses a hill climbing algorithm that gradually improves a multiple seed by swapping a 1 with a * in order to reduce as much as possible the overlap complexity. In this section we give a faster algorithm for this hill climbing heuristic. Assume a multiple seed *S *= {*s*_1_,*s*_2_,..., *s*_*k*_} each of weight *w *and denote the length of *s*_*i *_by ℓ _*i *_, for 1 ≤ *i *≤ *k*. We shall construct, for each pair (*i*,*j*) with 1 ≤ *i *≤ *j *≤ *k*, an ℓ_*i *_× ℓ _*j *_matrix OM_*ij *_defined by

$$\text{O}{\text{M}}_{ij}\left[r\right]\left[q\right]=\left\{\begin{array}{cc} 1, & \text{if}\;s_{i}\left[r\right]=s_{j}\left[q\right]=1,\\ 0, & \text{if}\;s_{i}\left[r\right]=s_{j}\left[q\right]=0,\\ -1, & \text{otherwise}. \end{array}\right)$$

**Figure 3:**
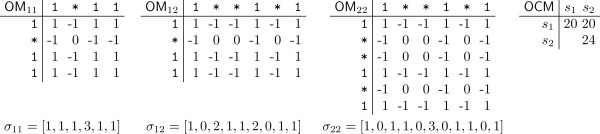
**An example of the data structures used in the new algorithm for hill climbing**. The matrices OM and OCM and the *σ *arrays are given for the seeds *s*_1 _= 1 * 11 and *s*_2 _= 1**1*1

We also consider an array *σ*_*ij *_of size ℓ_*i *_+ ℓ_*j*_- 1 defined as follows. The two seeds *s*_*i *_and *s*_*j *_can overlap in ℓ_*i *_+ ℓ_*j*_- 1 ways, each identified by the distance *r *between the right end of *s*_*j *_and left end of *s*_*i*_; we have 0 ≤ *r *≤ ℓ_*i *_+ ℓ _*j*_- 2. We then define σ_*ij*_[*q*] to be the number of positions where both seeds have a 1 for the *r*th overlap. We shall also store a *k *× *k *matrix OCM where OCM[*i*][*j*] = OC(*s*_*i*_, *s*_*j*_), for all 1 ≤ *i *≤ *j *≤ *k*. An example of the above data structures is shown in Figure 3.

We notice that the value of *o*_*ij*_[*q*] is obtained by counting the number of 1's in a NW-SE diagonal of OM_*ij*_; precisely, the positions considered are OM_*ij*_[*r*][*t*] with *r*-*t *= *q *- ℓ_*j *_+ 1.

**Figure 4:**
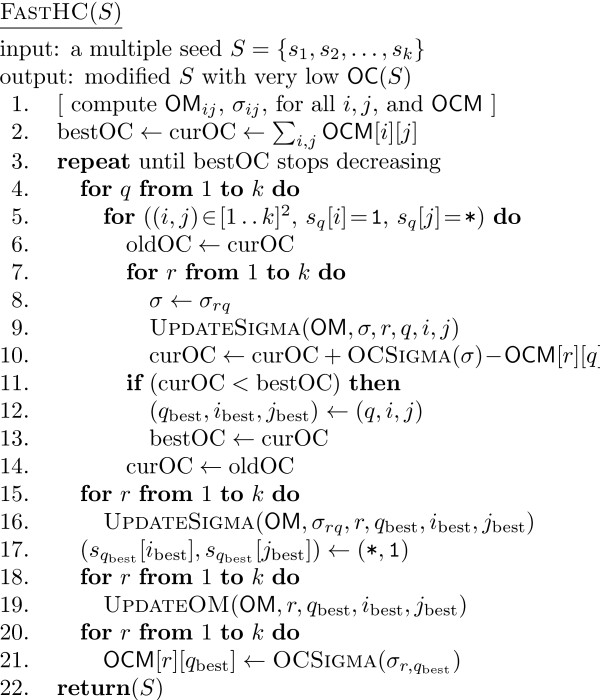
**The pseudocode of FastHC**. The pseudocode of the new faster algorithm for the hill climbing heuristic, FastHC

**Figure 5:**
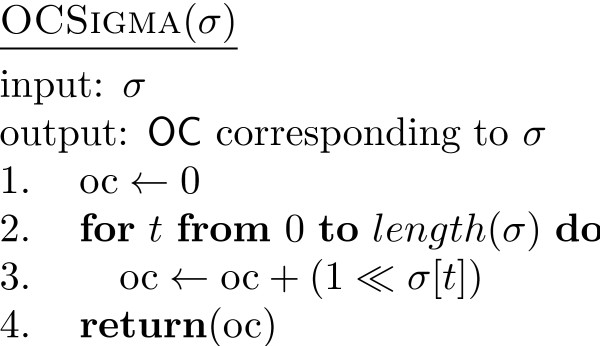
**The pseudocode of OCSigma**. The pseudocode of the additional function OCSigma, used by the main function FastHC.

**Figure 6:**
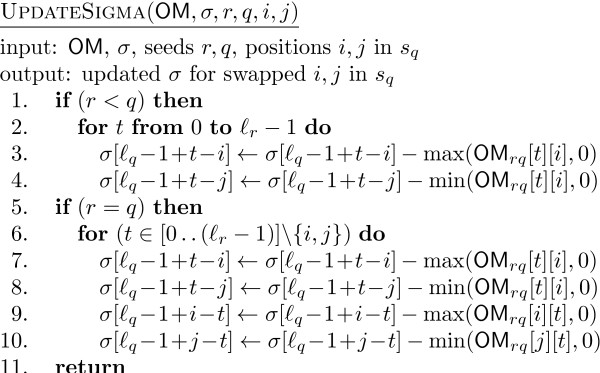
**The pseudocode of UpdateSigma**. The pseudocode of the additional function UpdateSigma, used by the main function FastHC

**Figure 7:**
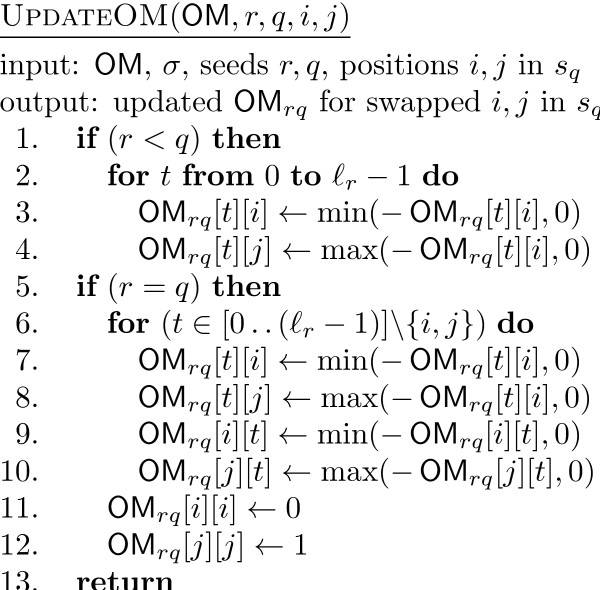
**The pseudocode of UpdateOM**. The pseudocode of the additional function UpdateOM, used by the main function FastHC

The new algorithm for hill climbing, FastHC, works as follows; the pseudocode is shown in Figure 4 with the additional functions given in Figures 5, 6, and 7. First, all matrices and arrays as above are computed according to their definitions (step 1). Then, for all seeds *s*_*q *_(step 4) and all pairs of positions in each with a 1 (at position *i*) and a * (at position *j*) (step 5), the potential reduction in OC is computed (steps 7-10) without actually changing the OM matrices or σ. The UpdateSigma procedure computes in linear time the new σ obtained assuming a swap between positions *i *and *j *in *s*_*q*_. If a better OC than the current best is obtained, the current seed and positions are stored together with the new best OC (steps 11-13). Either way the previous OC is restored (step 14). Once the best swap is found, everything is updated: σ arrays (steps 15-16), seeds (step 17), OM matrices (steps 18-19), and OCM matrix (steps 20-21). The σ arrays are updated before the seeds in order to be able to use the same UpdateSigma procedure, that works without the actual swap being performed.

To simplify the code, we employ the convention that any time we use *σ*_*rq*_, OM_*rq*_, or OCM[*r*][*q*], we assume *r *<*q*, otherwise, we would use *q, r *instead.

A difference needs to be made in both UpdateSigma and UpdateOM between the case *r *<*q *and *r *= *q*, since in the former the swap affects only one seed whereas in the latter it affects both. Notice also that the value of max(OM_*rq*_[*t*][*i*],0) is 1 when (OM_*rq*_[*t*][*i*],0) = 1 and 0 otherwise. Therefore, only when the previous value was 1 we subtract 1 from the appropriate component of σ. The value min(OM_*rq*_[*t*][*i*],0) is -1 only when OM_*rq*_[*t*][*i*] = -1 and, since a swap would cause this -1 to become a 1, the corresponding component of σ is incremented. Two other values, min(-OM_*rq*_[*t*][*i*],0) and max(-OM_*rq*_[*t*][*i*]), behave similarly. For the code of OCSigma it is sufficient to observe that (1 ≪ *σ*[*t*]) = 2^σ[*t*]^.

## Results

The new implementation of the hill climbing heuristic is one order of magnitude faster than the current implementation in SpEED. The results are summarized in Table 2, for a variety of parameters.

**Table 2:** Comparison of hill climbing algorithms

*w*	*N*	*p*	*k*	[ℓ_1_..ℓ_*k*_]	HC	FastHC
11	64	.70	16	[14..27]	7.79	**1.24**

22	50	.85	10	[25..37]	10.79	**1.35**

28	100	.90	8	[36..56]	39.83	**3.64**

28	150	.90	8	[39..63]	69.14	**5.86**

28	200	.90	8	[41..70]	108.74	**8.49**

28	100	.90	16	[33..59]	471.51	**41.42**

28	150	.90	16	[36..66]	788.79	**62.50**

28	200	.90	16	[39..72]	1075.10	**79.51**

Speed comparison between the existing (HC) and the new implementation (FastHC) of the hill climbing heuristic. Several sets of parameters are used. The times (in seconds) are given for a single multiple spaced seed with the given parameters. FastHC is up to 13.5 times faster than HS. Also, the improvement increases with the size of the input.

We apply the new implementation to compute better seeds for PatternHunterII and BFAST. The results are presented in Tables 3 and 4. The new seeds are better than those computed by Mandala, Iedera, and SpEED. For comparison we present also the single seeds of the same weight, both contiguous and spaced. The single spaced seeds in the case of BLAST have been computed also using FastHC.

**Table 3:** Sensitivity comparison of computed spaced seeds for PatternHunter

*w*	*N*	*p*	BLAST	PH	PHII	Mandala	Iedera	SpEED	FastHC
			(contig.)	(spaced)	(16 seeds)				

11	64	0.70	30.0196	46.7122	92.4114	92.3811	92.0708	93.2526	**93.3406**

11	64	0.75	49.4494	69.5844	98.4289	98.4320	98.3391	98.6882	**98.7156**

11	64	0.80	71.3993	88.2070	99.8449	99.8448	99.8366	99.8820	**99.8859**

Sensitivity comparison with Mandala, Iedera, and SpEED (results from [18]). Seeds were computed with the same parameters as those of PatternHunter II. FastHC (sensitivity values in bold) is the best in all cases. The sensitivity of the original seeds is significantly improved.

**Table 4:** Sensitivity comparison of computed spaced seeds for BFAST

*w*	*N*	*p*	1 seed (contig.)	1 seed (spaced)	BFAST (16 seeds)	Mandala	Iedera	SpEED	FastHC
22	50	0.85	14.4649	26.8064	58.6907	--	60.1535	60.8127	**60.9329**

22	50	0.90	36.6940	57.9846	87.3359	--	87.9894	88.5969	**88.7120**

22	50	0.95	74.1153	90.8265	99.2249	--	99.2196	99.3659	**99.3959**

Sensitivity comparison with Mandala, Iedera, and SpEED (results from [18]). Seeds were computed with the same parameters as those of BFAST. FastHC (sensitivity values in bold) is the best in all cases. The sensitivity of the original seeds is significantly improved.

Finally, we have computed several very heavy multiple seeds, using the weight of the default seed of MegaBLAST; see Table 5. We notice the low sensitivity of the MegaBLAST contiguous seed. Even at similarity 90% and length of the similar region sought for 200, the sensitivity is only around 67%. We have computed sets of 1, 2, 4, 8, and 16 seeds with the given weight 28, similarity 90% and *N *∈ {100, 150, 200}. The improvement in sensitivity over MegaBLAST is very large. Whereas the sensitivity of the MegaBLAST seed for the given parameters ranges from 39% to 67%, we need 16, 2, and 1 seeds, respectively, to reach sensitivities over 95%. All new seeds we computed are given in the Additional file 1 newSeeds.pdf.

**Table 5:** Sensitivity comparison of computed spaced seeds of MegaBLAST weight

*w*	*N*	*p*	MegaBLAST	1 seed	FastHC			
			(contig.)	(spaced)	2 seeds	4 seeds	8 seeds	16 seeds

28	100	0.90	39.1436	69.3241	79.6629	87.5674	92.7762	**95.9170**

28	150	0.90	55.4870	87.6426	93.4308	**97.0118**	98.7430	99.5137

28	200	0.90	67.4412	94.9876	**97.8936**	99.2937	99.7877	99.9409

Using FastHC, we computed multiple seeds with the same weight as the default seed of MegaBLAST for similarity 90% and *N *∈ {100, 150, 200}. The sensitivities of the new seeds are much higher than those of MegaBLAST. The values in bold show that the new 16, 2 and 2 seeds, respectively, reach sensitivities over 95% for *N *equal to 100, 150, and 200, respectively.

## Discussion

We have provided a much faster implementation of the hill climbing heuristic of SpEED, the leading software for computing multiple spaced seeds in the Bernoulli model. Using the new implementation, some of the most challenging seeds have been improved and new, even more difficult ones, were provided. Still, many problems remain open in this important area. A modified heuristic is needed to be able to compare seeds of different lengths, as well as to address models different from Bernoulli.

## Availability and requirements

Project name: SpEEDfast

Project home page: math.ryerson.ca/~silvana/SpEEDfast.cpp

Operating system(s): Platform independent

Programming language: C/C++

Other requirements: none

License: GNU GPL

Any restrictions to use by non-academics: none

## Abbreviations

BLAST: Basic local alignment search tool; OC: Overlap complexity.

## Competing interests

The author declares that they have no competing interests.

## Authors' contributions

SI designed and implemented the algorithms, performed the experiments, and wrote the manuscript. All authors read and approved the final manuscript.

## Availability of supporting data

The data sets supporting the results of this article are included within the article (and its Additional file 1).

## Supplementary Material

Additional file 1 **This file contains the new seeds computed using the improved heuristic**. Click here for file
