# Supplementary material for: Efficient computation of spaced seeds
Source: BMC Res Notes. 2012 Feb 28;5:123. doi: 10.1186/1756-0500-5-123 (PMC3392737; doi:10.1186/1756-0500-5-123)
Supplement: Additional file 1 — This file contains the new seeds computed using the improved heuristic. [file 1756-0500-5-123-S1.PDF]

Efficient Computation of Spaced Seeds – supplementary material

Silvana Ilie

PatternHunterII

similarity 70%
```
111*11*111*111
11*11*1*11***1111
1111**11****11*111
111*1*1**11*1**1*11
111*1***1*1**1*1**111
11*11**1***1***1*1*111
111***11*1***1**1***111
11*1*1**1*1*****1**11*11
111**1****1**1***1**1*111
11*11****1***11*****1*1*11
111**1**1**1******11**1*11
111**1*1****1*1****1**11*1
11*1*1***1*****1*1***1**111
11*1***1***1****1*1*1***111
111***1*1*****1**1****1*1*11
11*1*1**1***1*****1****11*11
```

similarity 75%
```
1111*1*111*111
111*11***11*1*111
1111**11*1**1*111
111**1**1**11**1111
111***1*1*1***11*111
111*1**1****11*1***111
111**1*1***1**1***11*11
11*11***1***1***1*11*11
11*11**1*1****1**1*1*11
11*11*1****1****11**1*11
11*1*1**1***1**1****11*11
111**1*1***1****1***1*1*11
111*1***1*****1**1**1***111
111*1*****1**1****1****1111
11*1*1****1**1******1*1*1*11
111***11*******1*1****1**111
```

similarity 80%
```
111*1*11*111*11
11*111**1**1*1111
1111***1*1*11***111
111*11****11***11*11
111***1*11***11*1*11
11*1**11**1***1*1*111
1*11*1**1*1***1*11*11
11*1*1*1****11**1**111
111**11****1**1***1*111
111*1****1**1**1*****11*11
11*1**1******1**11***1*111
111**1****1*1****1*1***111
11*1**1***1*1*****1***1*1*11
11*1*1***1****1****1**1**111
111**1*1*******1****1***1111
111*1***1***1***1****1*1**11
```

BFAST

similarity 85%
```
11111*1111*1111*111*111111
111*11*11111*1*111*1**111*1111
1111*111**11*11***1*11111*11111
11111*1*11**11*11*1111**1*11111
1111*11*1***11*11111**1**1*11*1111
11111**111111****11*1*1*1**11*1111
1111*111**1*1*1*11**1*1**111***11111
11111*1**111****11****1111*1*1*1*1111
1111**111*1*1**1****11**11**11*11*1111
111111**1**1*11*1*1*1**1***1**111*1111
```

similarity 90%
```
111111*1111*11111*1111111
11111*11*1*11*11**111*1*111111
1111*111*1*111*111***11*11*1111
11111*1**11111****1*11*1*111*1111
11111**11**1***1111*111**1*11*1111
1111**1111*1*1**1***1*111*1*1**11111
11111*1*111***111**1**1**11**11*1111
111*11*111****1**11*1*1*1***111**11111
1111*11***1*111**1**111***1**1*1*11111
11111*1*1***11**1*11****1*11**11*11111
```

similarity 95%
```
111111*1111*11*11111*11111
1111111*111*1*11***11*11*11111
1111*1*1*111**1*111*111**111111
11111*11****111111*11*1*1*11*111
111*1*11*1*11**11111***1*1*111*111
111111**11*11**1*1**111***11**11111
11111*1**11***11*11*1**11*1***111111
11111**11*1*11*****1***1*1*11*111*1111
11111*1**1*1***11****11*11**111*1*1111
1111*111*1***111*1***1***11*1**1*11111
```

BFAST weight – single seeds

similarity 85%
```
11111*111**111*1*11*11*111111
```

similarity 90%
```
11111*111**111*1*11*11*111111
```

similarity 95%
```
111111**111*1*11*1**11*111*1111
```

MegaBLAST weight – 1, 2, 4, 8, 16 seeds

$p = 90\%, N = 100$
11111*11*1*11**11*11***1111**1*1*1*111*1111
$p = 90\%, N = 150$
11111**111**11**1*1*11**1*1*11*1**11**1*111111
$p = 90\%, N = 200$
1111*11**111*1*1*1**111***1*11*1**11**11*11111

$p = 90\%, N = 100$
111111*1*111*1111*111*11*11*111*1111
111111*111**11**11*1**1*11*1*111*1*111111
$p = 90\%, N = 150$
111*11*1**1*111*1111***1*111*111*11*11111
11111*1*1111**11**11*1111***11*1*11*11111
$p = 90\%, N = 200$
1111*1*11111**1**1*11**111*111*1*1111*111
11111*11***1*111*111*11*1*11*111**1*11111

$p = 90\%, N = 100$
1111111*11111*1111*11111*1111111
111111*11*1*11*111**1***11111**1*1*111111
1111*111*1**11**1*1*1111*11*111**1111*111
11111*1*111***111*11*11*1***1*111111*1111
$p = 90\%, N = 150$
111*11*11111**111*1*11*111*11*1111111
111*1**111**1**1111111*1*1*111**111*1111
1111111*1111*11**1**11***111*1*111*1*1111
1111111***1*1*1111**1*1111*11*11*1**11111
$p = 90\%, N = 200$
11111*111*11111*1*1111**11*1111*1111
11111*11**111111*1*111**1*1**1*11**111111
1111111*1*11*1**11**1111*111*1***111*1111
11111**111***1*11*1*1**11*111111*11*11111

$p = 90\%, N = 100$
11111*1*111*111*1111*1111*111*11111
111**111111*1*1111*11**1*11111*111*111
1111*1111*11111***11**1*11*1*11111*1111
1111111**1*1111*11**111*1**111*11*1*1111
1111*11*111*1*11*11*1*1*111***11*11*11111
11111*11*111**1*1***1111111*11***1*111111
1111*1*11**11**11111*1*111**1**1111*11111
111*111*1**1**11*11111***111111*1*11*1111
$p = 90\%, N = 150$
1111*11111***111*1*11*11*111*11111111
1111*11*1**1111*111*11111*11*1*1*11111
11*111*11111111*1**1*111**1*1111**11111
1111*11**11111*1*11111**111*11*11**1111
1111*1*1**111*11**1*1**11111111**1111*111
11111*1111**1**11111***1*1*111*111**11111
11111**1**11*1*1111*1111*1***1111*11*1111
11111*1111*1*11**11*111*111*1**1*11*1*111
$p = 90\%, N = 200$
111111*111**111111111*1*11*11*1*1111
111111*11111*1**1*11*1111111*111*111
111*1*11*11111111**111*1*1111*111111
1111111*1***1*111*1**11*11111**1*111*1111
1111*111**1111**1111*1***1**11111*11*1111
11111*11111*11*1*1*111**1*11**11***111111
1111*1*11111**1**11***111*11*1*1111*11111
11111*1**1*1*11*1**111111**1111**11*11111

$p = 90\%, N = 100$
111111*1111*11111*11111*11111111
111*1*111111*11*111111111**1111*111
11111*11111*11111***11*111*111*11111
1111*111*111111*1111*1***1111*111111
1111111***11*1111*111*1111*11111*111
111111*11111*1*1*11*11*11*111**111111
1111*111*11**1**1111*1111111*1*1*11111
111111*11*1111**1**1111*1*1*1**11111111
11111*1111**1*111**1*1111*11*11*11*1111
11111**111*111*11111*1**111**1**1*111111
1111*11*11*1111111*1***11*1*11*1*11*1111
1111*1*11*111*11****111**111*11*1111*1111
1111111**1**1*111111*111*1**1**111*1*1111
1111*111*1*1***111*11*1***1111111**111111
11111*11***11*1**11***1*11111111*11*11111
11111*1111*1*11***111*111****111*1*111111

$p = 90\%, N = 150$
111*11111*11111*1*11111*11*1111111
11111*11111111111111**111*1*11*1111
1111111*111**11*111111*1111*111111
11111*1*1*11*11111*11*111111111*111
1111*11*11111111*11***111*111*11*1111
11111**1111*11*1**111111111*111**1111
11111*11*1*111*111*1111*11*1**1*111111
11111**11111***1111111*1*111*1**11*1111
1111111*11*11*111**1*1**11*111*1*1*11111
1111*111*1111***1111**11**1**11111*11111
11111*11*1***1*1111**1*1111*1**11111*1111
11111*111****11*1*111111*111**1*1*1*11111
11111*1*11*1*1*11*111***1*1*11111**111111
11111*11**111*11*1**111***1111***11111111
111111*1*1*11*11**1**11111***11*111*11111
1111*111111**1*1*1*1**1**1111*1111*1111

$p = 90\%, N = 200$
1111111*11*111111*1111*111*111111
1111*111*111*1*111111*11111111111
111111*11111111111*1*111*111*1111
11111*1*1111**11*1*11111111*11*11111
1111*1*111*1111*11**111*11111**111111
11111*1111***11*11111*1*1**11111*11111
11111*111*11111***11*11*1*11*1*1111*111
1111111***1*111*11**11111*1**11*1111*111
1111*111*1*11*11*11111**11*1*1**11*11111
1111*11*111**1111111**1*11*1***11111*111
111111**11**1*1***1*1111*1111111*1*11111
11111**11111*1*1111*11*11**11**1*1*11111
11111*1111*11**111****111111*1***11*11111
1111111*1**1**11111*11****111*111*1*11111
111*1111*1111*1****1*1**11111*11*111*1111
11111*111*1111*1*111***11****1111*11*1111
